# Inclusion of zero total event trials in meta-analyses maintains analytic consistency and incorporates all available data

**DOI:** 10.1186/1471-2288-7-5

**Published:** 2007-01-23

**Authors:** Jan O Friedrich, Neill KJ Adhikari, Joseph Beyene

**Affiliations:** 1 Interdepartmental Division of Critical Care, University of Toronto, Toronto, Canada; 2 Critical Care and Medicine Departments, St. Michael's Hospital, 30 Bond Street, 4Bond-015, Toronto, Ontario M5B 1W8, Canada; 3 Department of Critical Care Medicine, Sunnybrook Health Sciences Centre, 2075 Bayview Avenue Toronto, Ontario M4N 3M5, Canada; 4 Department of Public Health Sciences, University of Toronto, Toronto, Canada; 5 Child Health Evaluative Sciences, Hospital for Sick Children Research Institute, 123 Edward Street, Room 1206, Toronto, Ontario M5G 1E2, Canada

## Abstract

**Background:**

Meta-analysis handles randomized trials with no outcome events in both treatment and control arms inconsistently, including them when risk difference (RD) is the effect measure but excluding them when relative risk (RR) or odds ratio (OR) are used. This study examined the influence of such trials on pooled treatment effects.

**Methods:**

Analysis with and without zero total event trials of three illustrative published meta-analyses with a range of proportions of zero total event trials, treatment effects, and heterogeneity using inverse variance weighting and random effects that incorporates between-study heterogeneity.

**Results:**

Including zero total event trials in meta-analyses moves the pooled estimate of treatment effect closer to nil, decreases its confidence interval and decreases between-study heterogeneity. For RR and OR, inclusion of such trials causes small changes, even when they comprise the large majority of included trials. For RD, the changes are more substantial, and in extreme cases can eliminate a statistically significant effect estimate.

**Conclusion:**

To include all relevant data regardless of effect measure chosen, reviewers should also include zero total event trials when calculating pooled estimates using OR and RR.

## Background

A meta-analysis of randomized controlled trials of a particular treatment quantitatively synthesizes all available trial data and provides the least biased estimate of that treatment's effect. Results from individual studies are statistically combined to provide a weighted average estimate of overall treatment effect. In one common weighting scheme known as the inverse variance method, the treatment effect from each study is weighted by the inverse of its variance. For binary outcomes, the pooled effect measure is usually expressed as either a difference of proportions (risk difference, RD), a ratio of proportions (relative risk, RR), or a ratio of the odds (odds ratio, OR) of intervention and control group patients experiencing an event. The variances of the effect measures for individual studies are determined by two parameters: the number of study subjects in each group, and the number of these subjects with outcome events. Standard meta-analytic procedures also produce an estimate of heterogeneity, which is the extent of variability in treatment effects of individual trials beyond what would be expected by chance. For example, *I*^2 ^is one summary measure of heterogeneity and is the proportion of total variation among trials due to between-trial variation [1]. Using the inverse variance method of pooling studies, the weight of each study can be adjusted in the presence of significant heterogeneity by using the random-effects model [2].

In many studies of patients at low risk of developing the outcome that the intervention is designed to prevent, no subjects in either the intervention or the control group, or both, experience an outcome event. Meta-analyses using RR or OR as the effect measure traditionally include only studies with zero events in either the intervention or the control group, but not both. In contrast, meta-analyses using RD as the effect measure include studies with zero events in either *or *both groups [3,4].

The main argument for excluding zero total event trials (trials with zero events in both treatment and control arms) when the pooled effect measure is RR or OR is that they make no contribution to the magnitude of the treatment effect [5,6]. Although these trials do not contribute to producing a pooled treatment effect greater or less than nil, they do provide relevant data by showing that event rates for both the intervention and control groups are low and relatively equal [7]. Excluding such trial data potentially creates the risk of inflating the magnitude of the pooled treatment effect. Indeed, others have argued that zero total event trials should be included "to take into account the samples sizes of these studies" [8]. In addition, some published meta-analyses have included zero total event trials applying the standard continuity correction of 0.5 [9], which is commonly used for trials with zero events in only one arm [10,11]. Others have proposed different continuity corrections that perform better when the numbers of patients in the intervention and control groups are severely imbalanced [5].

The continuity correction is added to each cell of a 2 by 2 table for trials with zero events in one or both arms. When combining such trials using the inverse-variance method, continuity corrections are required to calculate individual trial effect estimates and variances (OR and RR) or to calculate individual trial variances (RD). Continuity corrections are less important or not required for other methods used for fixed effects analyses that do not incorporate between-study heterogeneity, such as Mantel-Haenszel methods and Peto OR [3-5]. Continuity corrections are also not required using Bayesian approaches that incorporate heterogeneity, although Bayesian methods require estimates of prior probabilities that are often based on subjective assessments and opinion [12].

Given these conflicting opinions and practices, the objective of this study was to examine, using illustrative examples, the influence of zero total event trials when the inverse variance method is used to pool RD, RR, and OR treatment effects.

## Methods

We selected 3 illustrative published meta-analyses that demonstrated a range of proportions of zero total event trials (among all trials with outcomes data), treatment effects, and heterogeneity [13-15]. For each binary effect measure, the meta-analysis was conducted including and excluding zero total event trials using the inverse variance random-effects model. For individual trials with no events in one or both groups, a continuity correction of 0.5 was added to each cell for each effect measure, as implemented in Review Manager 4.2 [4]. Calculations were carried out using standard equations and confirmed with Review Manager where possible.

## Results

**Figure 1:**
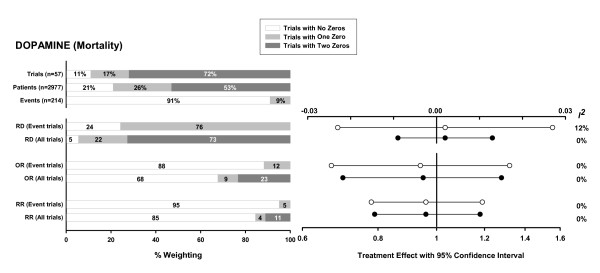
Meta-analysis investigating the use of low-dose dopamine to prevent or treat renal dysfunction [13]. Endpoint: mortality (total of 109 events in 1523 patients in the intervention group and 105 events in 1454 patients in the control group). The first three horizontal bar charts show the proportion of trials, patients, and outcome events in the meta-analysis of all "Trials with No Zeros" (trials with at least one event in both the intervention and control arms), "Trials with One Zero" (trials with no events in either the intervention or the control arm), and "Trials with Two Zeros" (trials with no events in both the intervention and control arms). The next three pairs of bar charts show the total weighting of all trials with no zeros, one zero, and two zeros, respectively, in the final pooled RD, OR, and RR. The first bar in each pair refers to a meta-analysis that includes only trials with at least one event in either the intervention or the control arm ("Event trials"). The second bar includes both trials with and without events ("All trials"). For each effect measure, a corresponding plot indicates the pooled treatment effect estimate with its 95% confidence interval (white circles when event trials only are included and black circles when all trials are included), and the *I*^2 ^heterogeneity measure. The RD is plotted on a linear scale, and OR and RR are plotted on the same logarithmic scale. Abbreviations: RD = risk difference, OR = odds ratio, RR = relative risk.

For the meta-analysis of low-dose dopamine [13] (Figure 1), the proportion of zero total event trials is large (>70%). Given the absence of a significant treatment effect among trials with at least 1 event, the inclusion of the zero total event trials decreases the confidence intervals of the pooled effect estimates but not the estimates themselves. This decrease in confidence interval is greatest for RD.

**Figure 2:**
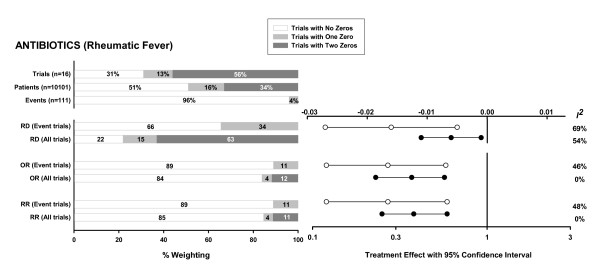
Meta-analysis investigating the use of antibiotics to treat sore throat [14]. Endpoint: acute rheumatic fever within 2 months of treatment (total of 37 events in 5656 patients in the intervention group and 74 events in 4445 patients in the control group). For a description of bar charts and plots see figure legend for Figure 1.

For the meta-analysis of antibiotics to prevent rheumatic fever [14] (Figure 2), the proportion of zero total event trials is smaller but still >50%. Including the zero total event trials reduces the magnitude of the treatment effect. However, the smaller treatment effect remains statistically significant, in part because of a simultaneous decrease in the width of the confidence interval resulting from the inclusion of more trials. As with the first example, the reduction in pooled treatment effect is greatest for RD. In this example, heterogeneity is either reduced or eliminated for each of the effect measures.

**Figure 3:**
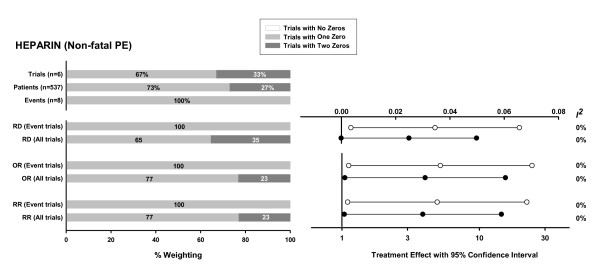
Meta-analysis investigating the use of prophylactic subcutaneous unfractionated or low-molecular weight heparin after hip fracture repair to prevent deep vein thrombosis [15]. Endpoint: non-fatal pulmonary embolism (total of 8 events in 245 patients in the intervention group and 0 events in 292 patients in the control group). For a description of bar charts and plots see figure legend for Figure 1.

In the third example (Figure 3) of heparin to prevent non-fatal pulmonary embolism [15], all trials have at least one arm with zero events, but only a third are zero total event trials. In this extreme example, inclusion of the zero total event trials also decreases the treatment effect for each pooled effect measure. However, the effect on RD is substantial and causes the statistical significance (2-sided p < 0.05) of the pooled RD to be lost.

## Discussion

Trials in which no patient develops the outcome of interest are conventionally included in estimates of pooled RD, but not RR and OR. We explored this inconsistency using 3 published meta-analyses and found that when zero total event trials are included, there is a relatively small reduction in the magnitude of the pooled RR and OR and the confidence intervals, resulting in a slightly more conservative estimate of treatment effect. In contrast, for RD where zero total event trials are traditionally included, the effect is more pronounced, and in 1 extreme case, inclusion of zero total event trials negated an otherwise statistically significant treatment effect. Therefore, excluding zero total event trials could change the clinical implication of a meta-analysis that used RD exclusively to pool similarly extreme data.

The greater effect of zero total event trials on RD versus RR and OR occurs because trials with low event rates have a much higher weight in the pooled estimate of RD compared to the other measures (graphically depicted in reference [16]). Because the pooled RR and OR is dominated by trials with at least 1 event in both groups, it is relatively insensitive to the inclusion of low-weight zero total event trials. Even when significant heterogeneity is present, which increases the relative weighting of low-weighted zero total event trials in random effects analyses, the changes in these pooled estimates are still relatively small (see Figure 2).

We present an extreme example (Figure 3) where the inclusion of zero total event trials in a meta-analysis using RD as the effect estimator negates a statistically significant treatment effect obtained when such trials are excluded. However, such situations would be expected to occur rarely because the inclusion of these trials has opposite impacts on the treatment effect (which becomes closer to nil) and its confidence interval (which narrows). For RR and OR, where the changes are smaller, it is even more unlikely that inclusion of zero total event trials would negate the statistical significance of a treatment effect, especially when the meta-analysis contains trials with at least 1 event in both groups.

The addition of zero total event trials decreased heterogeneity in the examples provided. One might have expected that heterogeneity would be increased by adding zero total event trials to a group of trials, each with at least 1 event, that on average show a non-zero treatment effect. However, in these examples the treatment effect was similar between zero total event trials and event trials. Therefore, the net effect of including more trials in the meta-analysis was to reduce heterogeneity. This arises because the Q statistic, which provides an assessment of heterogeneity, has a null Chi-squared distribution with degrees of freedom equal to one less than the number of trials. If heterogeneity increases only slightly as more trials are added, the increase in Q is small relative to the increased degrees of freedom and Q is less likely to be statistically significant. Similarly the *I*^2 ^measure of heterogeneity (calculated as 100%·[Q-degrees of freedom]/Q) decreases. Figure 2 illustrates this effect. Prior to the addition of the zero total event trials, there is a significant treatment effect and substantial heterogeneity for each of the effect measures. After adding the zero event trials, the treatment effect is smaller but still significant, and the degree of heterogeneity, expressed as *I*^2^, is reduced or eliminated for each of the effect measures.

For the RD effect measure, the weighting of the zero total event trials in the pooled result is comparable to the proportion of such trials in the meta-analysis. Therefore, including a large proportion of zero total event trials with identical results (i.e. a RD of exactly 0) may result in reduced heterogeneity by simply overwhelming the other results even if there is marked heterogeneity among the remaining trials. Even in the presence of equal underlying event rates in the treatment and control groups one would expect some random variation around a RD of 0; however, zero total event trials are frequently small which makes it less likely to observe any events in either arm. Thus, these small underpowered studies contribute to an exaggerated decrease in heterogeneity. One would expect this to be less of a factor for OR and RR because the weighting of zero total event trials in the effect measure is lower, and sometimes significantly lower, than the proportion of such trials included in the meta-analysis (for example see figures 1 and 2).

We used a continuity correction of 0.5 since this is the correction most commonly used [3,4]. Sweeting et al [5] have recently proposed two alternative continuity corrections, one based on the reciprocal of the group (i.e. treatment or control) size opposite the zero cell, and the second based on an empirical estimate of the pooled effect size using the studies in the meta-analysis with events in both the treatment and control arms. Applying these corrections instead of 0.5 in two of our examples (dopamine and antibiotics to prevent rheumatic fever) gives very similar results. For the third extreme example (heparin to prevent non-fatal pulmonary embolism), there are no trials with events in both the treatment and control groups, preventing the use of the second alternative correction. Inclusion of zero total event trials using the continuity correction based on the reciprocal of the opposite group size negates a statistically significant treatment effect obtained when such trials are excluded for all three effect estimators [similar to RD in Figure 3, the lower bounds of the 95% confidence interval for the RR and OR effect estimators also cross unity if this alternative correction is used (results not shown)]. Using this correction in our case examples shows that including or excluding zero total event trials can change the clinical implication of a meta-analysis pooling similarly extreme data, regardless of the effect measure used.

We focused on the impact of zero total event trials on summary effect measures in meta-analyses using inverse variance weighting, which is the only commonly used method that can incorporate between-study heterogeneity in a random effects model. We chose published illustrative meta-analyses that combined high- and low-risk patients. We did not consider other issues such as the choice of effect measure, appropriateness of combining high- and low-risk patients, the choice of a fixed vs. random effects model, or other methods for addressing heterogeneity.

In contrast to our illustrative examples combining high- and low-risk patients, two recently published simulation studies provide comprehensive comparisons of multiple meta-analytic methods when baseline event rates are low [5,17]. These studies, both of which excluded zero total event trials for the OR simulations, suggest that in general the commonly used continuity correction of 0.5 biases the Mantel-Haenszel and inverse variance OR estimators [5,17]. The simulation study that examined RD demonstrated that when events are rare, using RD widened confidence intervals and thus lowered statistical power compared to other methods [17]. Finally, although the inverse variance method is the only non-Bayesian method that incorporates between-study heterogeneity, the same authors found that it gives biased effect estimates when event rates are low. Although this bias is present at moderate event rates and effect sizes, it becomes greater than 1% when event rates are very low (± 1%) or treatment effects very large (RR ± 0.5) [17]. The overall event rates in our examples were 7.7% [13], 1.1% [14], and 1.5% [15], respectively.

## Conclusion

In summary, the exclusion of zero total event trials from meta-analyses increases the effect size compared to meta-analyses that include these trials. The magnitude of this increase is relatively small for RR and OR, but more substantial if RD is used to pool the results. Zero total event trials are currently included only if RD is used as the effect measure. We believe these trials should also be included if RR or OR are the effect measures to provide a more conservative estimate of effect size (even if this change in effect size is very small for RR and OR), and to provide analytic consistency and include the same number of trials in the meta-analysis, regardless of the summary effect measure used. Inclusion of zero total event trials would enable the inclusion of all available randomized controlled trial data in a meta-analysis, thereby providing the most generalizable estimate of treatment effect.

## Abbreviations

OR – odds ratio

RD – risk different

RR – relative risk

## Competing interests

The author(s) declare that they have no competing interests.

## Authors' contributions

JF was involved with the conception and the design of the study, acquisition, analysis and interpretation of data, and drafted the manuscript. NA and JB were each involved with the design of the study, analysis and interpretation of data, and critical revision of the manuscript for important intellectual content. All authors read and approved the final manuscript.

## Pre-publication history

The pre-publication history for this paper can be accessed here:


